# The effect of traditional Chinese medicine treatment for post-viral olfactory dysfunction: A protocol for systematic review and meta-analysis

**DOI:** 10.1097/MD.0000000000025536

**Published:** 2021-04-23

**Authors:** Fangfang Ma, Hewei Zhang, Bingxue Li, Peiyu Cheng, Yunfei Ma, Mingwei Yu, Xiaomin Wang

**Affiliations:** a Department of Oncology, Beijing Hospital of Traditional Chinese Medicine, Capital Medical University, Dongcheng District, Beijing; b The First Affiliated Hospital of Soochow University, Suzhou; c Beijing University of Chinese Medicine, North of the Third Ring, Chaoyang District, PR China.

**Keywords:** COVID-19, efficacy, meta-analysis, olfactory dysfunction, post-viral

## Abstract

**Background:**

Post-viral olfactory dysfunction (PVOD) have been reported in infections caused by several respiratory viruses, especially in COVID-19 which influence severely the quality of life of affected subjects. Few study has been published on the treatment of PVOD. Traditional Chinese medicine (TCM) is an effective method for PVOD which effects and safety have been confirmed. Therefore, this study is aim to evaluate the effects of TCM on PVOD.

**Methods:**

A searching strategy will be carried out mainly in the following databases in English and Chinese, PubMed, EMBASE, Cochrane Central Register of Controlled Trials, China Network Knowledge Infrastructure (CNKI), Chinese Scientific Journal Database (VIP), Chinese Biomedical and Medical Database (CBM), and Wanfang Database. Only randomized controlled trials related to TCM for PVOD will be included to enhance effectiveness. The primary outcome is the effective rate of PVOD. The secondary outcomes are included olfactory domain value examination, visual analogue scale (VAS), questionnaires of olfactory disorders (QOD), T&T olfactometer test, Sniffin ticks test, and any other clinical assessments. Two authors will independently perform study selection, data extraction, and quality assessment to ensure the quality of the systematic evaluation. Every disagreement will be deal with by the third author. Data synthesis and subgroup analysis will be performed in the Review Manager V 5.3.3.

**Results:**

This study is aim to evaluate the efficacy and safety of TCM in PVOD.

**Conclusion:**

: This meta-analysis may provide more reliable evidence-based medical evidence for clinical practice to assist patient in relieving PVOD.

**Ethics and dissemination:**

There is no need to acquire ethical approval for individuals come from literatures instead of recruiting directly. The findings of this review will be reported in peer-reviewed publications and/or presented at relevant conferences.

**Prospero registration number:**

CRD42021238977.

## 1 Introduction

The World Health Organization (WHO) has confirmed >115 million cases of COVID-19 worldwide, with a total of 115,198,906 cases and 2,555,854 deaths before March 3, 2021. COVID-19 may become a global or local epidemic of infectious diseases as other viral illness like influenza. Clinical manifestations of COVID-19 range from mild, cold-like symptoms typically associated with respiratory tract infections, such as cough and fever, to severe pneumonia with respiratory failure.^[1,2]^ Infected patients may face long-term sequelae, repeated infections, repositive, and prolonged or intermittent incubation periods.

Frequently, patients also experience smell and taste disorders (STD).^[3–9]^ These mainly consist of a decrease or loss of smell (hyposmia and anosmia) and taste (hypogeusia and ageusia); alterations in the chemesthesis—that is, the chemical sensitivity of mucosa to irritants-; and/or variations in the quality of chemosensory perception (phantosmia and parosmia).

The proportion of COVID-19 subjects experiencing STD is considerable, around 41% and 62% according to 2 recent meta-analyses.^[10,11]^ With objective evaluations of STD used, the proportion of COVID-19 patients with olfactory alterations was 73% to 98%, which is considerably higher than 44% by subjective evaluation.^[5,12,13]^ 89% of patients has a complete resolution of STD after 4 weeks from diagnosis,^[14]^ with 10 days of median duration. However, more prolonged course could be possible. One-third of patients has reported only a partial improvement of STD 40 days after diagnosis while 5% of patients reported no improvement.^[15]^ We observed a high percentage of persistent smell dysfunction at 6 months from the diagnosis of SARS-CoV-2 infection, with 11.7% of patients being anosmic or severely microsmic. These data highlight a significant long-term rate of smell alteration in patients with previous SARS-COV-2 infection.^[16]^

Post-viral olfactory dysfunction (PVOD) have been reported in infections caused by several respiratory viruses, including coronaviruses.^[17,18]^ They usually follow the onset of respiratory symptoms and are associated with inflammatory changes in the respiratory mucosa and mucous discharge.^[19,20]^ A loss of olfactory sensory neurons due to dysfunction of supporting cells, inflammation-related apoptosis, or possibly direct infection could be hypothesized in patients showing slow recovery from of STD.^[21]^

PVOD could influence severely the quality of life of infected patients with long-term lasting, and even be linked with depression.^[22,23]^ The treatment is challenging and few explored in COVID-19. The glucocorticoids and olfactory training has been reported with some benefit,^[24,25]^ but no data available on the efficacy in post-viral olfactory disorders.

Olfactory disorders has been recorded with the pathogenesis of lung-related for >2000 years in the classic of traditional Chinese medicine (TCM), “The Yellow Emperor's Internal Canon of Medicine.” TCM has accumulated rich literature and cases, among which the treatment methods mainly include TCM decoction, acupuncture, acupoint injection, and the combination of various means.^[26]^ As a promising method for olfactory dysfunction, TCM has the advantages of internal and external treatment, definite clinical curative effect, and no obvious adverse reactions.^[27–31]^

At present, there are few studies on the treatment of PVOD. Therefore, a meta-analysis is used to analyze the results of relevant clinical trials and evaluate the effects of traditional Chinese medicine on PVOD, so as to, provide more reliable evidence-based medical evidence for clinical practice.

## 2 Methods

### 2.1 Study registration

This protocol will be prepared according to recommendations of the Preferred Reporting Items for Systematic review and Meta-Analysis Protocols. It was registered on PROSPERO (CRD42021238977).

### 2.2 Search methods for study identification

Relevant studies will be searched in the following databases from inception to March 1, 2021, in English and Chinese: PubMed, EMBASE, Cochrane Central Register of Controlled Trials, China Network Knowledge Infrastructure (CNKI), Chinese Scientific Journal Database (VIP), Chinese Biomedical and Medical Database (CBM), and Wanfang Database. The major search terms are (“Traditional Chinese medicine” OR “Chinese medicine” OR “Chinese herb” OR “decoction” OR “acupuncture” OR “moxibustion” OR “massage” OR “cupping” OR “nasal irrigation”) AND (“viral illness” OR “post-viral” OR “virus” OR “viral”) AND (“olfactory disorders” OR “olfactory dysfunction” OR “smell disorder” OR “cacosmia” OR “dysosmia” OR “paraosmia” OR “anosmia”). Additionally, more information will be searched as possible from the following sources: unpublished conference proceedings and ongoing trials from the World Health Organization International Clinical Trials Registry Platform (http://apps.who.int/trialsearch/) and Current Controlled Trials (http://www. controlled-trials.com).

### 2.3 Criteria for study selection

#### 2.3.1 Types of studies

Only randomized controlled trials (RCTs) will be included in this systematic review with the diagnosis of PVOD. Observational studies, case reports, and animal studies will be excluded.

#### 2.3.2 Types of participants

This study will consider patients at any age with olfactory dysfunction attributed to viral illness regardless of their race and sex. Additionally, patients with olfactory loss attributed to chronic auto-immune disease, chronic neurodegenerative disease, head trauma/traumatic brain injury, or iatrogenic causes will be excluded.

#### 2.3.3 Types of interventions

##### 2.3.3.1 Treatment interventions

In the treatment group, the intervention will be the comprehensive treatment of Traditional Chinese medicine combined with/without conventional treatment. We will include the studies that evaluate any type of Traditional Chinese medicine. These included studies can perform various forms of Traditional Chinese medicine (Traditional Chinese medicine decoction, acupuncture, moxibustion, massage, cupping).

##### 2.3.3.2 Control interventions

In the control group, the patients will receive conventional treatment (western medicine or/and olfactory training) that prevent PVOD.

#### 2.3.4 Types of outcome measures

The primary outcome will be the effective rate of PVOD in the treatment. The secondary outcomes will include olfactory domain value examination, visual analogue scale (VAS), questionnaires of olfactory disorders (QOD), T&T olfactometer test, Sniffin ticks test, and any other clinical assessments. All reported side effects and adverse events will be included as safety outcomes.

### 2.4 Data collection and analysis

#### 2.4.1 Selection of studies

**Figure 1:**
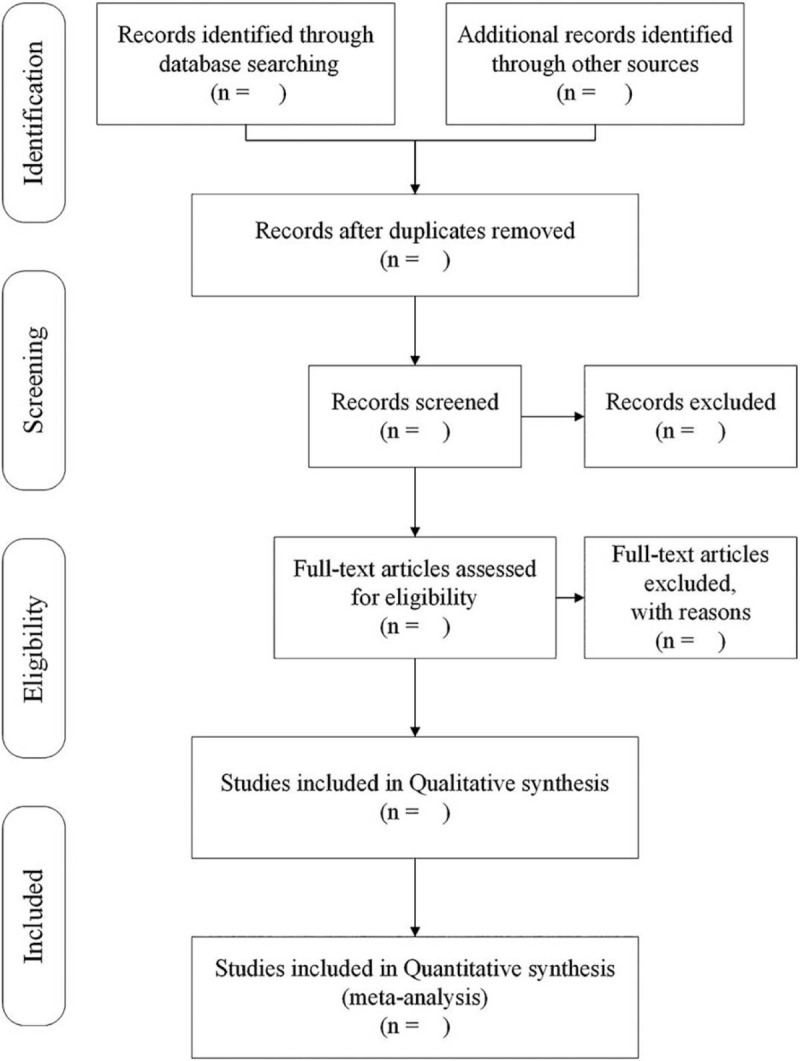
Flow diagram of this study selection.

All the authors will be trained regarding the purpose and process of the review. Three independent authors will perform the selection work. The study selection will be responded by 2 authors by screening the titles, abstracts, and even the full texts of all included studies independently to decide whether it should be potentially eligible studies. All the studies meeting eligibility criteria will be therefore included in the review with documenting in an Excel spreadsheet with details of study name, author, publishing year, country, and database. Meanwhile during screening abstract and full-text evaluation, the spreadsheet will show out the reasons for inclusion and exclusion. If any disagreement exists throughout the process, the third author will be required on making the final decision. The selection process of eligible papers is shown in a Preferred Reporting Items for Systematic Review and Meta-analysis (PRISMA) flow diagram (Fig. 1).

#### 2.4.2 Data extraction and management

After filtering the final eligible studies, the data will be extracted independently by 2 reviewers. Two independent reviewers will conduct the data extraction from the eligible study and fill in the data collection sheet. The third author will adjudicate any disagreements during the process. According to the recommendations of the Cochrane Handbook, all extracted data and information management will be put into an extraction form Microsoft Excel.

The following data will be extracted:

1. Basic characteristics of studies: title, first author's name, year of publication, country, and the journal.
2. Participants’ characteristics: age, sex, number of participants, disease, inclusion criteria, exclusion criteria, baseline situation.
3. Interventions: details of TCM (such as treatment methods, sessions, frequency), treatment duration, study design, randomization, allocation concealment, and blinding methods.
4. Comparators: western medicine or/and olfactory training.
5. Outcomes: measures, primary, and secondary outcomes.

#### 2.4.3 Assessment of risk of bias

The Cochrane Collaboration “Risk of bias” assessment tool will be used to assess the potential sources of bias in the included studies. Two independent authors will first evaluate the risk of bias of eligible studies separately and then cross-check their findings. This quality assessment will be based on random sequence generation, allocation concealment, blinding of participants and personnel, blinding of outcome assessment, incomplete outcome data, selective reporting, and other bias. We will grade bias risk of the included trials and classify them into 3 levels as “high risk,” “low risk,” and “unclear risk.” The third author will resolve any disagreement and make a final decision.

#### 2.4.4 Measures of treatment effect

The available data of treatment outcomes will be extracted and meta-analyzed. Weighted mean difference (MD) or standardized mean difference (SMD) with 95% CIs will be used for continuous data. The risk ratio (RR) with its 95% CI will be used for dichotomous data.

#### 2.4.5 Dealing with missing data

If the presented data of the studies are not consistent or missing, we will first try to contact the corresponding author or relevant author for the required data by email. Otherwise these studies will be excluded without obtaining missing data.

#### 2.4.6 Data synthesis

RevMan software (V.5.3) will be used to complete the data analysis and synthesis by the Cochrane Collaboration. The forest plots and the heterogeneity between the included studies will be performed in the software. If little significant heterogeneity exist among the trials, a fixed effect model will be established while a random effect model will be carried out for significant heterogeneity. Dichotomous data will be analyzed by risk ratio (RR) with 95% CIs, otherwise, continuous data by mean difference (MD) or standard mean difference (SMD) with 95% CIs.

#### 2.4.7 Assessment of heterogeneity

The statistical heterogeneity will be assessed in the forest plot and detected by standard *X*^2^ test and *I*^2^ test. The interpretation of the *I*^2^ is as follows: 0% to 40%: might not be important. 30% to 60%: moderate heterogeneity. 50% to 90%: may represent substantial heterogeneity. 75% to 100%: considerable heterogeneity.

#### 2.4.8 Assessment of reporting biases

If the meta-analysis includes >10 trials, funnel plots will be generated to analyze the potential publication bias. Egger test will be used to evaluate quantitative analysis.

#### 2.4.9 Subgroup analysis

If obvious heterogeneity exists in a single meta-analysis, subgroup analyses will be conducted to analyze the heterogeneity of available data according to variations in characteristics of trial participants, types of traditional Chinese medicine, and type of conventional treatment.

#### 2.4.10 Sensitivity analysis

Sensitivity analysis is needed to evaluate the robustness and reliability of the results when sufficient data exist with obvious heterogeneity. We conducted the sensitivity analysis in 2 ways: exclude any of the study. Change the effect model to verify the result synthesized. When a low-quality study is identified and excluded, the meta-analysis will be pertained to low the heterogeneity or not. The certain result can be compared with determine whether the low-quality study should be included. The final result will depend on the sample size, missing data, risk of bias, and quality of methods of each study.

#### 2.4.11 Certainty assessment

The quality of the evidence will be assessed with GRADE system as 4 levels: very low, low, moderate, and high. Two investigator will assess independently and give a summary of finding table together. The third investigator will resolve any disagreement and make a final decision.

## 3 Discussion

There are 3 major causes of olfactory disorders as nasal sinuses inflammatory diseases, viral infections, and head trauma. PVOD has accounted for 18.60% to 29.30% among all outpatients with olfactory disorders.^[32]^

Studies have shown that the human olfactory nervous system can be reshaped. Olfactory sensory neurons in the olfactory mucosa have the characteristics of sustainable regeneration lifetime in the nervous system of all vertebrates. Olfactory training can repeatedly stimulate the olfactory epithelium and olfactory pathway through various olfactory elements, so that the damaged olfactory function can be improved or restored.^[33–36]^ There are few effective way of western medicine to treat PVOD. The systemic glucocorticoid and ginkgo biloba extract have been recommended but the curative effect is uncertain.^[24]^ One studies have shown that the total effective rate of PVOD patients treated with nasal pneumatic spray aerosol inhaled budesonide suspension was 90%, but most of the patients (65%) did not return to normal.^[37]^

In one cohort of Wuhan in December 2019, which aimed to explore the frequencies of nasal symptoms in patients with COVID-19, only 1 out of 10 hospital admitted patients had loss of smell which was associated to severity of COVID-19. About 80% of COVID-19 patients recovered from smell and taste dysfunction in 2 weeks.^[38]^ Compared with other countries, the lower incidence and higher cure rate of PVOD patients with COVID-19 in Wuhan, seems to attribute to widely use of Chinese Medicine.

Traditional Chinese medicine has a large number of classic records in the treatment of “No smell of nose” in 2000 years. A wealth of treatment information has been accumulated, not only internal clothing but also various external treatment such as stuffing nose, blowing nose, nasal irrigation, acupuncture, massage, and so on. Acupuncture and moxibustion has been proven effective in olfactory disorder after virus infection,^[39]^ and improve the olfactory sensitivity of healthy people.^[40]^ The decoction of TCM, as “Danggui Shaoyao Powder” and “Ginseng Yangrong Decoction,” has been proven more effective than nasal glucocorticoid with 43% and 36% of patients respectively in improving olfactory disorders after viral infection.^[41]^

However, the number of studies to be included may be small. Because most of the clinical experience has been reported by case reports, TCM studies on PVOD have lower-level evidence for statistical analysis. Further research is needed for the principle of acupuncture and TCM decoction. In order to prove the effectiveness of the TCM treatment on PVOD, we may have included olfactory disorders patients of different reasons and this may enlarge the sample size to some extent.

## Author contributions

**Conceptualization:** Fangfang Ma, Hewei Zhang.

**Investigation:** Fangfang Ma, Bingxue Li.

**Methodology:** Peiyu Cheng, Yunfei Ma, Mingwei Yu.

**Writing – original draft:** Fangfang Ma, Hewei Zhang.

**Writing – review & editing:** Fangfang Ma, Hewei Zhang, Xiaomin Wang.
